# Extracellular Vesicle Subtypes Released From Activated or Apoptotic T-Lymphocytes Carry a Specific and Stimulus-Dependent Protein Cargo

**DOI:** 10.3389/fimmu.2018.00534

**Published:** 2018-03-15

**Authors:** Christine Tucher, Konrad Bode, Petra Schiller, Laura Claßen, Carolin Birr, Maria Margarida Souto-Carneiro, Norbert Blank, Hanns-Martin Lorenz, Martin Schiller

**Affiliations:** ^1^Division of Rheumatology, Department of Internal Medicine V, University Hospital Heidelberg, Heidelberg, Germany; ^2^Department of Infectious Diseases, Medical Microbiology and Hygiene, University Hospital Heidelberg, Heidelberg, Germany; ^3^Laboratory Dr. Limbach and Colleagues, Medical Care Unit, Heidelberg, Germany; ^4^ACURA Center for Rheumatic Diseases, Baden-Baden, Germany

**Keywords:** apoptosis, exosomes, extracellular vesicles, microvesicles, stimulus-dependent release

## Abstract

Extracellular vesicles (EVs) are released from nearly all mammalian cells and different EV populations have been described. Microvesicles represent large EVs (LEVs) released from the cellular surface, while exosomes are small EVs (SEVs) released from an intracellular compartment. As it is likely that different stimuli promote the release of distinct EV populations, we analyzed EVs from human lymphocytes considering the respective release stimuli (activation Vs. apoptosis induction). We could clearly separate two EV populations, namely SEVs (average diameter <200 nm) and LEVs (diameter range between 200 and 1000 nm). Morphology and size were analyzed by electron microscopy and nanoparticle tracking analysis. Apoptosis induction caused a massive release of LEVs, while activated T-cells released SEVs and LEVs in considerably lower amounts. The release of SEVs from apoptotic T-cells was comparable with LEV release from activated ones. LEVs contained signaling proteins and proteins of the actin-myosin cytoskeleton. SEVs carried cytoplasmic/endosomal proteins like the 70-kDa heat shock protein 70 (HSP70) or tumor susceptibility 101 (TSG101), microtubule-associated proteins, and ubiquitinated proteins. The protein expression profile of SEVs and LEVs changed substantially after the induction of apoptosis. After apoptosis induction, HSP70 and TSG101 (often used as exosome markers) were highly expressed within LEVs. Interestingly, in contrast to HSP70 and TSG101, gelsolin and eps15 homology domain-containing protein 3 (EHD3) turned out to be specific for SEVs irrespective of the stimulus causing the EV release. Finally, we detected several subunits of the proteasome (PSMB9, PSMB10) as well as the danger signal HMGB1 exclusively within apoptotic cell-released LEVs. Thus, we were able to identify new marker proteins that can be useful to discriminate between distinct LEV subpopulations. The mass spectrometry proteomics data are available *via* ProteomeXchange with identifier PXD009074.

## Introduction

Extracellular vesicles (EVs) are released from a variety of mammalian cells. These vesicles can be discriminated by size or molecular composition and two main EV populations have been described. To date, a population of large EVs (LEVs)—released from the cellular surface—is distinguished from a population of small EVs (SEVs), which is released from an intracellular/endosomal compartment, the multivesicular body. These EV populations have been termed microvesicles (considering LEVs) and exosomes (considering SEVs, respectively). Microvesicles are large vesicles with varying sizes in a diameter range from 200 up to 1,000 nm. These vesicles carry receptors and surface molecules from the cell of origin (1–6). The release of microvesicles results from reorganization of the actin-myosin cytoskeleton, and occurs by direct budding of the outer cellular membrane (7–9). Exosomes are vesicles smaller than 200 nm and are released from an intracellular/endosomal compartment, called multivesicular body (4, 10). Due to their endosomal origin exosomes carry endosome-associated proteins, such as Rab GTPases, Alix, or tumor susceptibility 101 (TSG101) (11–13). Exosomes and their molecular composition have intensively been studied and TSG101, the 70-kDa heat shock protein 70 (HSP70) or tetraspanins (e.g., CD63, CD81) have been reported as exosome marker proteins (14–19).

Beside analyses of their protein cargo, EVs have been reported to carry DNA or distinct RNAs and they are able to transfer genetic information from cell to cell (20–26). Thus, EVs are increasingly recognized as mediators of intercellular communication (27–30), and their role in the pathogenesis of autoimmune diseases or tumor growth has been discussed (3, 31–36). However, while the EV field is rapidly expanding, there is an urgent need to better define vesicle subpopulations. Meanwhile, the heterogeneity of EVs is a well-known fact and it has become evident that SEVs (often called exosomes) consist of diverse subpopulations. A diversity that applies for LEVs (often referred to as microvesicles or apoptotic bodies) as well (5, 17, 37).

The release of EVs can be triggered by many stimuli like cellular activation or apoptosis induction (5, 38). Thus, it seems obvious that different stimuli might favor the release of a distinct EV type. Recently, a first systematic study by Théry and co-workers analyzed and directly compared the protein content of exosomal and non-exosomal EVs released from primary human monocyte-derived dendritic cells was analyzed (39). Nevertheless, a comparative study, which systematically analyzes EVs, released after different stimulation conditions (i.e., cellular activation or apoptosis induction) is still needed. To address this issue, EVs were isolated from primary human T-lymphocytes. Activated T-lymphocytes were generated by PHA/IL-2 stimulation of PBMCs resulting in >95% CD3 positive T-cells (23, 40–42) (for details see Materials and Methods). These activated T-cells were then further stimulated with IL-2 or induced to undergo apoptosis by UV-B irradiation. LEVs were isolated by filtration following an ultracentrifugation at 10,000 × *g*. In a further ultracentrifugation step (100,000 × *g*), SEVs were isolated.

Both vesicle populations (SEVs and LEVs, respectively) were isolated either after cellular activation or after the induction of apoptosis. SEVs and LEVs were quantified, morphologically analyzed, and the protein content of each distinct EV population was investigated. We could show that apoptosis induction is the most potent stimulus for the release of LEVs. SEVs and LEVs were released in considerably lower amounts from activated T-cells. SEV release from apoptotic T-cells was comparable to LEV release from activated ones. Moreover, we demonstrated that the protein cargo of SEVs and LEVs is tightly regulated and dependent on the stimulus causing EV release, with apoptosis induction dramatically changing the protein cargo of both vesicle populations. As an example, when analyzing vesicles released from viable T-cells, TSG101 (a classically used exosome marker) is exclusively found within SEVs. After apoptosis induction, the same protein was detected within LEVs and was virtually excluded from the SEVs population. Similar changes were also observed, when we analyzed protein modifications, such as ubiquitination. By means of two dimensional difference gel electrophoresis (DIGE) followed by mass spectrometry, we were able to identify 24 proteins which are differentially expressed and regulated within SEVs and LEVs after cellular activation or apoptosis induction.

Finally, we have gained insight into the release mechanisms of EVs. We observed proteins of the actin-myosin cytoskeleton (actin, ezrin) within LEVs, whereas microtubule-associated proteins [gelsolin (GSN) or eps15 homology domain-containing protein 3] are exclusively and specifically found within SEVs. Importantly, these two proteins were specific markers for the SEV population even after the induction of apoptotic cell death. Our findings indicate that the release of LEVs is dependent on the activation of the actin-myosin cytoskeleton, suggesting a release from the cellular surface. SEVs, however, seem to be mainly released from inside the cell, and their release is associated with the microtubule apparatus. Furthermore, we observed an accumulation of proteasome subunits within LEVs and showed that the proteasome is involved in the regulation of LEV release. Interestingly, the inducible proteasomal subunits as well as the danger signal protein HMGB1 accumulated in LEVs released from apoptozing T-cells. These proteins can be used as new markers to identify distinct LEV subpopulations.

## Materials and Methods

### Cell Culture

Healthy donors were participated in this study after having given informed consent. The study, approved by the ethics committee of the University of Heidelberg, was conducted according to the ethics guidelines of our institution and those of the Declaration of Helsinki.

Peripheral blood mononuclear cells (PBMCs) were isolated by density gradient centrifugation (LSM 1.077, Merck, Darmstadt, Germany) out of heparinized venous blood from normal healthy donors. PBMCs were washed twice in phosphate-buffered saline (PBS, Sigma, Taufkirchen, Germany) and were cultured in RPMI 1640 (Life Science Technologies, Darmstadt, Germany) supplemented with 10% (v/v) heat inactivated fetal calf serum (Gibco-BRL, Eggenstein, Germany) 10 mM HEPES buffer, 4 mM l-glutamine and penicillin-streptomycin (Sigma, Taufkirchen, Germany). Cell viability was checked by trypan blue exclusion test. To generate activated T-lymphocytes 1 µg/ml phytohemagglutinine (Sigma, Taufkirchen, Germany) and 0.5 U/ml IL-2 (Roche, Mannheim, Germany) were added to the culture media for 5 days, leading to activation and marked proliferation of T-cells. These PHA/IL-2 activated lymphocytes are >95% CD3 positive (23, 40–44). Cells were then washed and stimulated with additionally 0.5 U/ml IL-2 to further expand T-cells.

### Isolation of LEVs and SEVs

Extracellular vesicles-depleted culture medium was prepared overnight by centrifugation at 100,000 × *g* in a 70Ti rotor (Beckman Coulter, Krefeld Germany). To isolate EVs, T-cells were washed with PBS and plated into cell culture dishes with up to 100 × 10^6 T-cells per 30 ml EV-depleted culture medium. T-cells were either activated by the addition of IL-2 or induced to undergo apoptosis by UV-B irradiation for 30 s (90 J/cm^2^). Afterward T-cells were cultured for 20 h, LEVs and SEVs were isolated by differential ultracentrifugation. Before EV isolation, cell vitality was always assessed by staining with AnnexinV (AxV, Böhringer, Mannheim, Germany) and propidiumiodide (PI, Sigma, Taufkirchen, Germany). In short, T-cells were incubated with 200 ng of AxV-FITC and 500 ng PI in 500 µl Ringer’s solution (B. Braun, Melsungen, Germany) for 30 min at 4°C and analyzed by flow cytometry. To deplete whole T-cells or apoptotic cellular remnants before EV isolation the cell suspension was centrifuged at 300 × *g*, 5 min and the remaining supernatant was passed through a 1.2 µm non-pyrogenic, hydrophilic syringe filter into ultracentrifuge tubes (Beckman Coulter). Subsequently, EVs were isolated by centrifugation at 10,000 × *g* for 45 min at 10°C in a 70Ti rotor (Beckman Coulter) to receive LEV pellets. The resulting supernatant was then centrifuged at 100,000 × *g* for 45 min at 10°C to receive SEV pellets. All pellets were resuspended in either 50 µl sterile PBS for nanoparticle tracking analysis (NTA) or in 10 µl RIPA-lysis buffer (c-c-pro, Neustadt, Germany) supplemented with protease inhibitors for protein analysis (complete Mini, Böhringer, Mannheim, Germany).

### Nanoparticle Tracking Analysis

A NS300 NTA machine (Malvern, Amesbury, United Kingdom) was used to analyze isolated EVs. Following camera settings were used: camera level 13/14, screen gain 1.0, and threshold 6. For each sample, a quick measurement for 60 s was performed to ensure right dilution factor and camera settings. Standard measurement was performed by taking at least three videos with 60 s, with more than 500 tracks per video.

### Immunoblot Analysis

Extracellular vesicles pellets were resuspended and lysed in RIPA-lysis buffer (c-c-pro, Neustadt, Germany) supplemented with proteases inhibitor cocktail (complete Mini, Böhringer, Mannheim, Germany) for 30 min on ice. Lysates were centrifuged by 16,000 × *g* for 10 min at 4°C. Afterward, the supernatant of each probe was transferred into a new microfuge tube and the protein concentration was quantified by BCA assay (Life Technologies, Darmstadt, Germany). 15 µg protein of each probe was diluted in loading buffer and loaded onto a 12.5% SDS-PAGE. After transfer to PVDF membranes, proteins were detected by using the following antibodies:

Rabbit polyclonal anti-human: Actin (Sigma, Taufkirchen, Germany), ERK1 (K-23), HSP90 (Santa Cruz, Heidelberg, Germany), LAT (Upstate Biotechnology, New York, NY, USA), PSMA1 (clone N1C3, Biozol, Eching, Germany), ZAP70 (Epitomics, Burlingame, Canada). Goat polyclonal anti-human: Ezrin (C-19), GSN (N-18) (Santa Cruz, Heidelberg, Germany), PSMB10 (Biotechne, Wiesbaden-Nordenstadt, Germany). Mouse monoclonal anti-human: pERK (E-4), EHD3, HSP70, Ubiquitination (Santa Cruz, Heidelberg, Germany), LCK (clone 28, BD Pharmingen, Heidelberg, Germany), anti-TSG101 (clone 4A10), PSMB9 (clone 792520, Biotechne, Wiesbaden-Nordenstadt, Germany). Blots were treated with species-specific horseradish peroxidase-labeled antibodies (Dianova, Hamburg, Germany) and signals were detected with enhanced chemiluminescence (Amersham Biosiences, Freiburg, Germany).

### Two Dimensional DIGE

Isolated EVs obtained from activated and apoptozing T-lymphocytes (three normal healthy donors) were lysed in THC buffer supplemented with 1 µg/ml Aprotonin, Leupeptin, Pepstatin, and 200 mM PMSF (Merck, Darmstadt, Germany). Lysates were sonicated (program: 1 s pulse and 5 s pulse off, 3 min, amplitude: 90%) two times at 4°C and centrifuged at 20,000 rpm for 15 min at 4°C. The supernatant was transferred into a new microfuge tube and protein concentration was determined by Bradford assay. Afterward each probe was divided into three parts with equal protein amounts of 5 µg. Each part was labeled with a specific dye for DIGE preparations (CYDYE DIGE Fluor CY3/CY5, VWR, Darmstadt, Germany). An internal standard was generated through mixing 5 µg protein of each probe and labeling with CY2 (CYDYE DIGE Fluor, VWR, Darmstadt, Germany). After labeling, probes were loaded onto pH stripes (VWR, Darmstadt, Germany) for the protein separation in the first dimension (isoelectric point). Then proteins were separated in the second dimension (molecular weight, standard SDS-PAGE). The experiments were performed in triplicates. Including the standards, 36 gels were obtained. To identify protein spots, 2D-gels were scanned (IPGphor3, GE Healthcare, Freiburg, Germany) and analyzed by a DIGE DeCyder machine and software (V7.0).

### Mass Spectrometry

After the analysis by the DIGE DeCyder machine and software (GE Healthcare, Freiburg, Germany) we selected 24 protein spots, for identification of the proteins by mass spectrometry. Selected protein spots were picked out of the SDS-gels using an Ettan spot picker (GE Healthcare, Freiburg, Germany) and analyzed by mass spectrometry (LC-MS/MS). Mass spectrometry was performed at the Core Facility for Mass Spectrometry in the ZMBH in Heidelberg. The mass spectrometry proteomics data have been deposited to the ProteomeXchange Consortium via the PRIDE (45, 46) partner repository with the dataset identifier PXD009074 and 10.6019/PXD009074.

### Transmission Electron Microscopy (TEM)

Isolated and pelleted EVs were fixed in glutaraldehyde-PBS (2% final). After washing and post-fixing in osmium tetroxide (2%) and K4[Fe(CN)6] (1.5%), samples were totally enclosed in contrast solution uranyl acetate, dehydrated with a graded dilution series of ethanol, and finally embedded into glycid-ether-100-based resin. Ultrathin sections were cut (Reichert Ultracut S ultramicrotome, Leica Microsystems). Slices were contrasted and analyzed with a Zeiss EM 10 CR electron microscope.

### Proteasome Inhibition Assay

Activated T-lymphocytes were induced to undergo apoptosis by UV-B irradiation for 30 s (90 J/cm^2^) in the presence or absence of 1 nM bortezomib (Santa Cruz, Heidelberg, Germany) or 10 µM Y27632 (Merck, Darmstadt, Germany). After 20 h, the amount of released LEVs was quantified by flow cytometry (FSC/SSC analysis), using an EPICS XL™ flow cytometer (Coulter, Hialeah, Fl, USA). Cell viability was assessed by AxV/PI staining as described above.

### Statistics

Paired two-tailed Student’s *t*-tests was used to perform statistical analysis. Experiments, in which cells of the same donor were treated with different conditions, and the respective experiment was repeated *n*-times with different donors, paired two-tailed Student’s *t*-tests was performed. All statistical analyses, excluding DIGE analysis, were performed using the statistical software GraphPad Prism 5.0 (GraphPad Software, Inc., San Diego, CA, USA).

Statistical analysis for DIGE analysis was performed using DIGE DeCyder software V7.0 (GE Healthcare, Freiburg, Germany). Statistical level of significance was determined as a *p*-value of <0.05.

## Results

### Morphologic Characterization of EVs Released From Activated or Apoptozing Human T-Lymphocytes

The morphology and molecular composition of EVs have intensively been studied since their discovery. However, vesicle preparations have been analyzed using different cellular models and distinct stimuli causing EV release (22, 47). So far, no study has analyzed different EV populations and simultaneously considered the respective release stimulus. Therefore, we characterized and compared different EV populations released either after cellular activation or after apoptosis induction.

**Figure 1:**
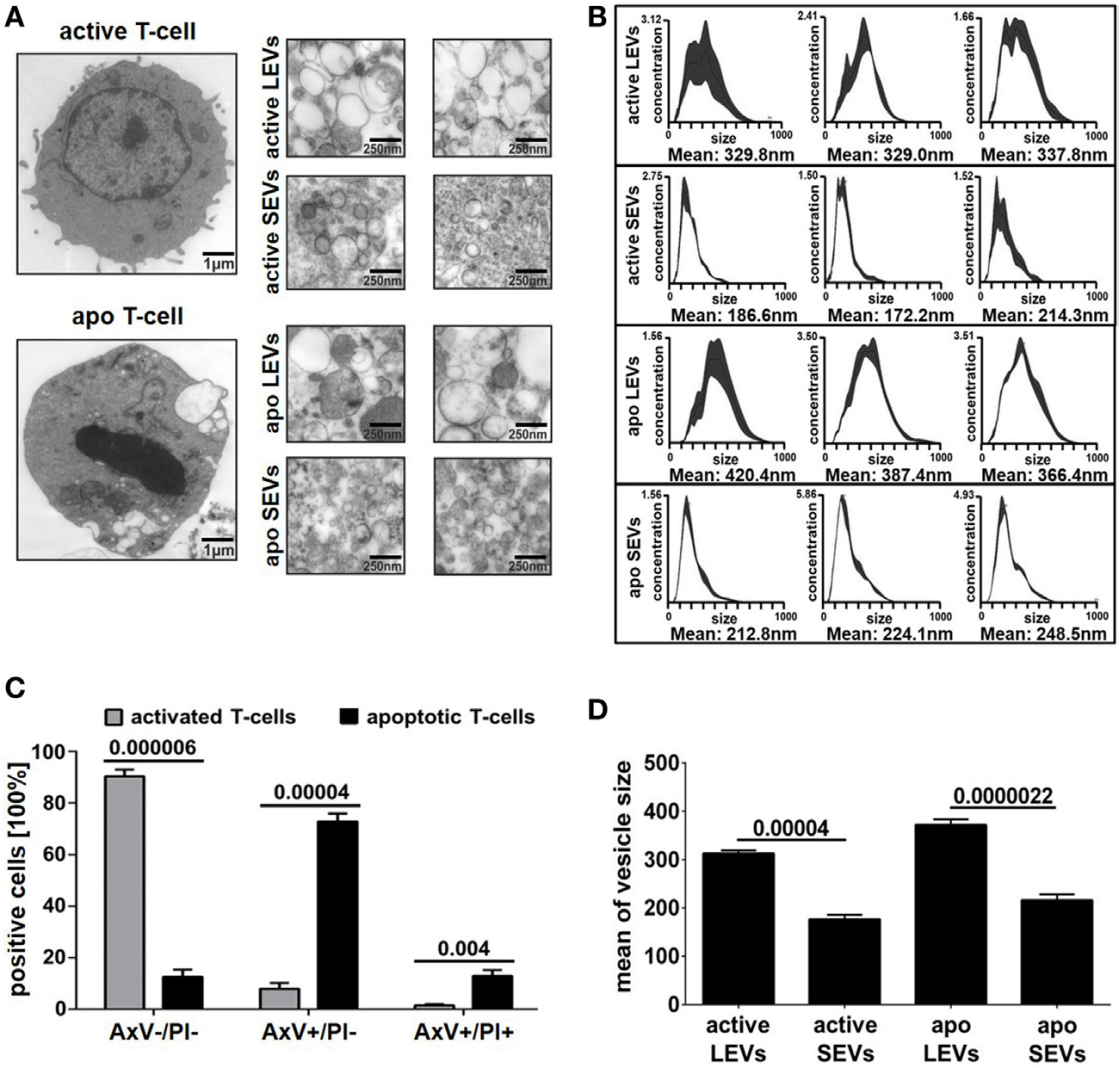
Morphology of large EVs (LEVs) and small EVs (SEVs). **(A)** Large pictures on the left show a representative T-cell either after cellular activation (upper picture) or after apoptosis induction (lower picture). The corresponding extracellular vesicles are shown on the right side (*active* LEVs: LEVs released from activated T-cells; *active* SEVs: SEVs released from activated T-cells; *apo* LEVs: LEVs released from apoptozing T-cells; *apo* SEVs: SEVs released from apoptozing T-cells). **(B)** Three representative size distribution graphs with the corresponding mean values obtained by nanoparticle tracking analysis (NTA) measurements are shown. On the *x*-axis, size distribution (nm) is depicted; the *y*-axis outlines vesicle concentration (10^6^ vesicles/ml). **(C)** The graph shows the percentage of viable T-cells (AxV+/PI−), apoptotic T-cells (AxV+/PI−), and necrotic T-cells (AxV+/PI+). Activated or UV-B irradiated T-cells were analyzed by flow cytometry after AxV/PI staining. Data were obtained from five independent experiments (mean values + SEM). **(D)** The graph shows mean values of vesicle size distribution (NTA analysis) obtained from 10 independent experiments. Mean values + SEM are shown. Statistical significance was calculated employing the Student’s *t*-test.

Large EVs and SEVs were isolated from the supernatant of activated or apoptozing primary human T-lymphocytes. After apoptosis induction, the amount of AxV+ positive and PI− negative T-cells was markedly increased (90% increase, *p* = 0.00004, Figure 1C). The percentage of necrotic T-cells (AxV+/PI+ positive ones) was only slightly increased (8.7% increase, *p* = 0.004, Figure 1C). The isolation of EVs was performed by filtration followed by differential ultracentrifugation. Collected EVs were analyzed by TEM, NTA, and BCA assay. TEM preparations showed LEVs, released from viable and apoptozing T-cells, as membrane enveloped vesicles in sizes from 200 to 1000 nm in diameter (Figure 1A). SEVs, released from viable and apoptozing T-cells, occurred as membrane-coated vesicles, but differed dramatically in size with an average diameter from 50 to 200 nm. Isolated EVs were also analyzed by NTA as shown in Figure 1B. For LEVs isolated from viable T-cells a mean size of 330 nm was calculated, whereas LEVs released from apoptozing T-cells appeared somewhat larger (100–750 nm) with an average size of 390 nm. In contrast, SEVs appeared as smaller vesicles with an average size of 190 nm for SEVs released from viable T-cells. Again, SEVs released after apoptosis induction appeared somewhat larger when compared to those released from activated T-cells.

**Figure 2:**
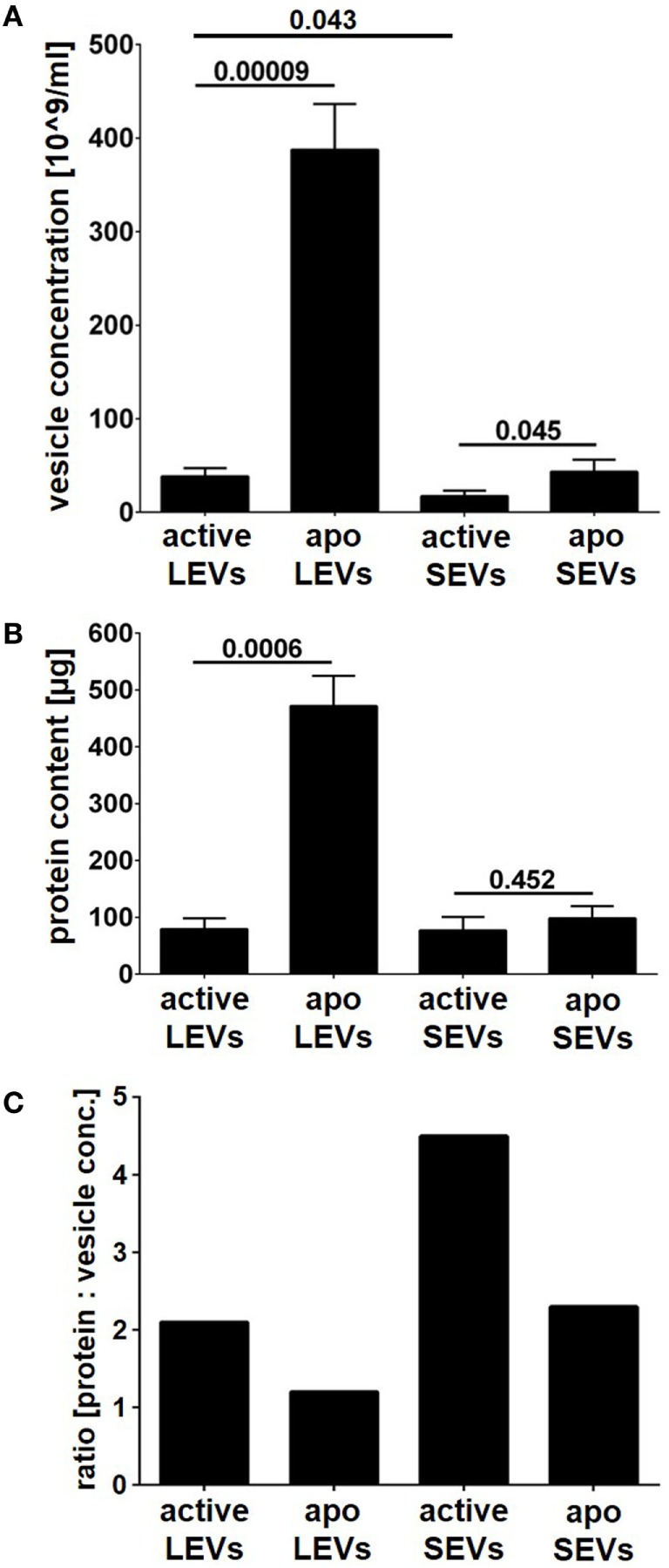
Vesicle concentration and protein content of large EVs (LEVs) and small EVs (SEVs). LEVs and SEVs were isolated after activation of T-cells or after apoptosis induction (*active* LEVs: LEVs released from activated T-cells; *active* SEVs: SEVs released from activated T-cells; *apo* LEVs: LEVs released from apoptozing T-cells; *apo* SEVs: SEVs released from apoptozing T-cells). **(A)** The concentration of isolated extracellular vesicles (EVs) was quantified by nanoparticle tracking analysis (NTA). **(B)** The total protein content of isolated EVs is shown. The graphs show mean values out of eight independent experiments + SEM. Statistical significance was calculated employing the Student’s *t*-test. **(C)** The graph shows the ratio calculated by division of protein content through vesicle concentration [protein content (μg) divided by vesicle concentration (×10^9^ vesicles/ml)]. *Active* LEVs: ratio score = 2.1; *active* SEVs: ratio score = 4.5; *apo* LEVs: ratio score = 1.2; *apo* SEVs: ratio score = 2.3.

Next, we compared the amount of vesicles (SEVs and LEVs) released either after cellular activation or after apoptosis induction. The release of LEVs was massively triggered after apoptosis induction. Here, the LEV concentration rose up to a 10-fold level from 38 to 388*10^9^ vesicles/ml (*p* = 0.00009, Figure 2A). Further, we observed a slight increase of SEVs released from apoptozing T-cells (*p* = 0.045, Figure 2A). Activated T-cells released slightly more LEVs when compared to SEVs (*p* = 0.043, Figure 2A). In parallel, we analyzed the amount of protein released within the different vesicle populations and obtained very similar results (Figure 2B). Further, a protein/vesicle ratio was calculated by division of protein content through the vesicle concentration (Figure 2C). The highest protein/vesicle ratio was found in SEVs released from activated T-cells (ratio score = 4.5, Figure 2C). Apoptotic SEVs and activated LEVs showed an equal protein/vesicle ratio (ratio score = 2.3, respectively 2.1, Figure 2C). The lowest protein/vesicle ratio was observed by the apoptotic LEVs (ratio score = 1.2, Figure 2C).

As suggested previously by the international society of EVs (48) we have analyzed a set of proteins, which either should be present in or excluded from distinct EV populations. Proteins associated with compartments other than plasma membrane or endosomes should not be detectable within SEVs. In fact, we were able to show, that the mitochondrial protein Bcl-2 is excluded from all EV fractions (LEVs and SEVs) and only present within whole cells. The proteins calnexin and calreticulin (endoplasmatic reticulum related) were detected in whole cells, to a lesser extent also in LEVs, but were excluded from the SEV fraction. Annexin I, a protein which is expected to be present within EVs (48), was in fact detected in whole cells as well as in all EV fractions (Figure S1 in Supplementary Material).

### Protein Profile of LEVs and SEVs Released From Activated or Apoptozing Human T-Lymphocytes

**Figure 3:**
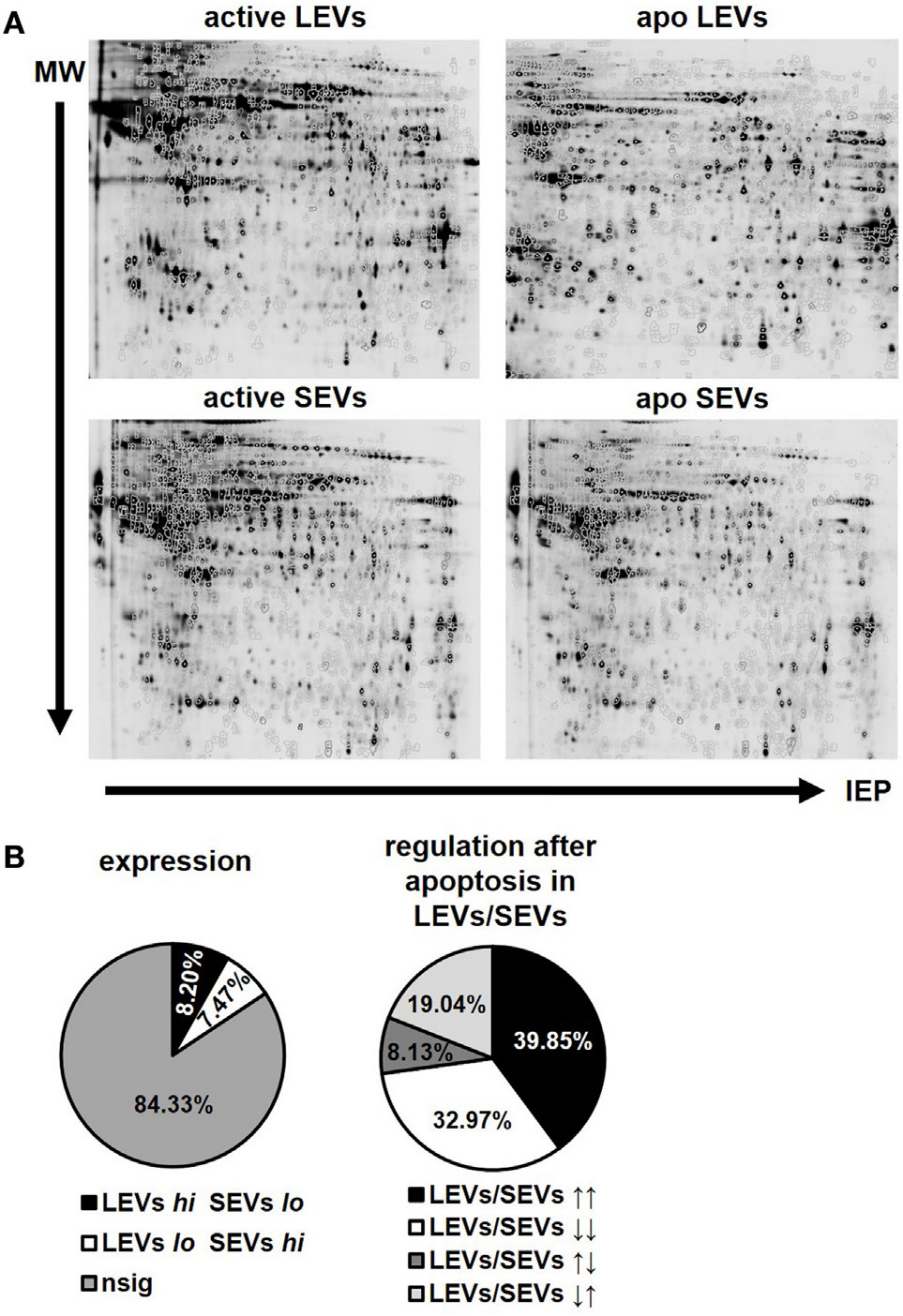
Protein expression and regulation pattern of large EVs (LEVs) and small EVs (SEVs) after apoptosis induction. **(A)** One representative gel is shown for bidimensional electrophoresis of activated and apoptotic LEVs and SEVs. **(B)** The pie charts on the left indicate the percentage of proteins showing a different expression level within LEVs or SEVs. LEVs *high* SEVs *low* is shown in black (8.20%), LEVs *low* SEVs *high* is shown in white (7.47%). Proteins showing no significant differences in their expression within the respective EVs are shown in gray (84.33%). The right pie chart indicates the percentage of proteins which have been differentially regulated after cellular activation or apoptosis induction. LEVs *high*/SEVs *high* is shown in black (39.85%); LEVs *low/*SEVs *low* is shown in white (32.97%); LEVs *high*/SEVs *low* is shown in dark gray (8.13%); LEVs *low*/SEVs *high* is shown in light gray (19.04%). Results are calculated out of three independent experiments and statistical significance (*p* ≤ 0.05) was calculated employing the Student’s *t*-test.

After the characterization of the morphology of EVs, we were interested in the proteins present in distinct vesicle populations. To this aim, we used two dimensional DIGE and western blot analysis. For DIGE analysis, SEVs and LEVs were isolated from apoptozing or activated T-cells. One representative gel for each condition (activated LEVs, apoptotic LEVs, activated SEVs, and apoptotic SEVs) is depicted in Figure 3A. Using the DIGE DeCyder 2D software we could map 11,364 protein spots (100%). We identi-fied 9,583 protein spots (84.33%) that did not significantly differ in their expression levels. However, 1,781 protein spots (15.67%) showed either a different expression level in SEVs and LEVs or a significant change in expression after the induction of apoptosis. To evaluate the expression profile in a more detailed way we divided the protein spots into two major categories:

(1) Spots showing a different expression level when comparing LEVs and SEVs. Based on a *p*-value ≤0.05 we found 930 spots that were significantly higher expressed within LEVs (8.20%), while 846 protein spots showed a higher expression in SEVs (7.47%, Figure 3B, left pie chart).
(2) Spots that were up- or downregulated within distinct EV populations after apoptosis induction. These spots were either regulated in parallel or in opposing directions when comparing LEVs and SEVs. Here, 701 protein spots showed a simultaneous upregulation in LEVs and SEVs (39.85%), while 580 protein spots were simultaneously downregulated in both vesicle populations (32.97%, Figure 3B, right pie chart). 143 protein spots were upregulated in LEVs and downregulated in SEVs (8.13%) and 335 spots were regulated *vice versa* (19.04%).

**Figure 4:**
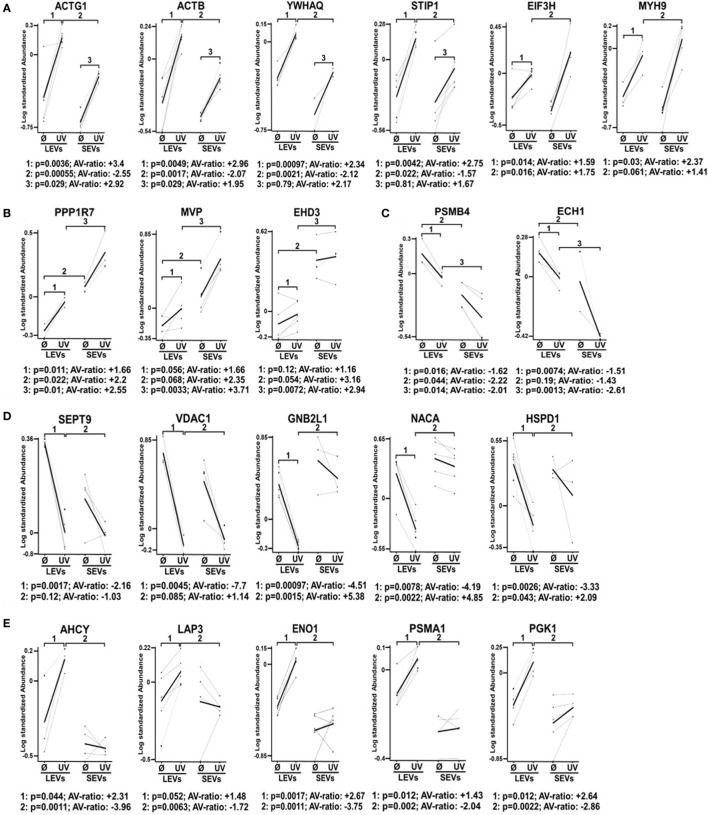

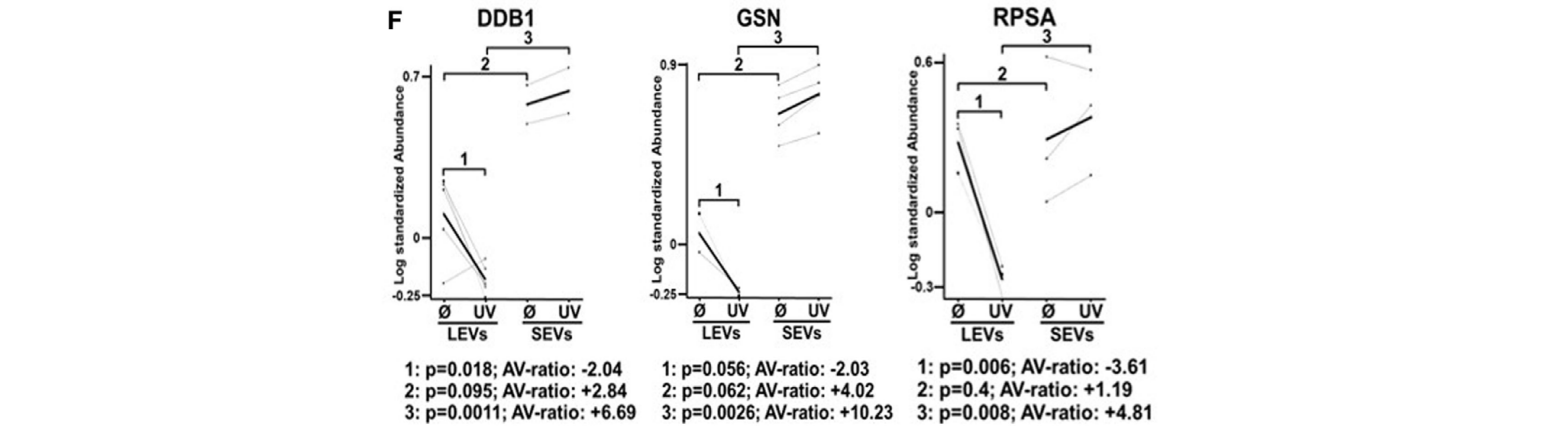
Differential expression pattern of proteins in large EVs (LEVs) and small EVs (SEVs) isolated after cellular activation or apoptosis induction. The graphs show the expression level of proteins that have been identified by mass spectrometry. Results obtained from analysis of the 2D-gels using the difference gel electrophoresis (DIGE) DeCyder machine and software are shown (standardized protein abundance on the *y*-axis). Protein expression levels of LEVs or SEVs isolated either after cellular activation (Ø) or after the induction of apoptosis by UV-B irradiation (UV) are indicated. **(A,B)** Expression levels are shown of proteins that have been upregulated in both extracellular vesicles (EV) fractions after the induction of apoptosis. **(C,D)** Graphs show proteins that have been downregulated in LEVs and SEVs. **(E,F)** Graphs show proteins with a different expression and regulation in LEVs and SEVs. EV preparations were obtained from three healthy donors. Statistical analysis (*p*-value and AV-ratio) was done using the DIGE DeCyder software.

Finally, we selected 24 protein spots which were either strongly regulated after the induction of apoptosis or which showed a significantly different expression in SEV and LEV preparations. Those spots were then identified by mass spectrometry (Figure 4).

Figure 4A shows proteins which were upregulated in LEVs and SEVs after apoptosis induction. Here we identified gammaactin (ACTG1), beta-actin (ACTB), 14-3-3 protein theta (YWHAQ), stress-induced phosphoprotein 1, eukaryotic translation initiation factor three subunit H (EIF3H), myosin heavy chain 9 (MYH9) (Figure 4A).

The proteins shown in Figure 4B were also upregulated after apoptosis induction, but showed different expression levels when comparing LEVs and SEVs. These proteins are: protein phosphatase 1 regulatory subunit 7 (PPP1R7), major vault protein, eps15 homology domain-containing protein 3 (EHD3).

The proteins shown in Figures 4C,D showed a downregulation after apoptosis induction. These proteins are: proteasome subunit beta type-4 (PSMB4), enoyl-CoA hydratase 1, septin 9 (SEPT9), voltage-dependent anion-selective channel 1, guanine nucleotide-binding protein subunit beta-2-like 1 (GNB2L1), nascent-polypeptide-associated complex alpha polypeptide (NACA), and the heat shock 60 kDa protein 1 (HSPD1).

Figures 4E,F show proteins, which are differentially regulated and expressed (comparing LEVs and SEVs) after apoptosis induction. The proteins are: adenosylhomocysteinase, leucine aminopeptidase 3, alpha-enolase 1, proteasome subunit alpha type 1 (PSMA1), phosphoglycerate kinase 1, DNA damage-binding protein 1 (DDB1), GSN, and the 40 S ribosomal protein SA.

**Figure 5:**
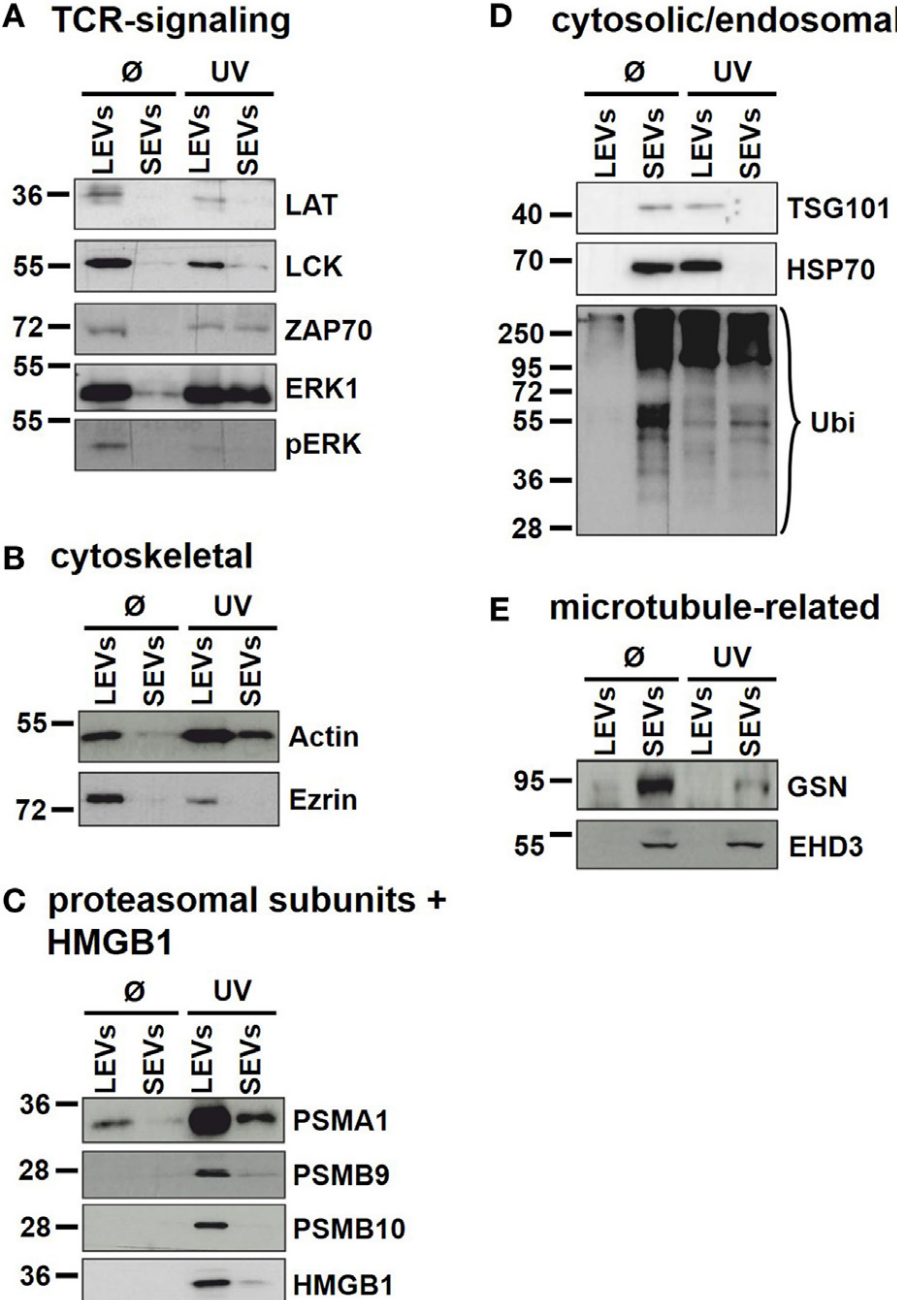
Immunoblot analysis of proteins present in distinct extracellular vesicles (EV) populations. Large EVs (LEVs) and small EVs (SEVs) were isolated from activated T-cells (Ø) and T-cells induced to undergo apoptosis (UV). Analyzed proteins included: **(A)** proteins related to the T-cell receptor signaling, **(B)** cytoskeletal proteins, **(C)** proteasomal subunits and HMGB1, **(D)** cytosolic/endosomal proteins, and **(E)** microtubule-related proteins.

Our findings demonstrate that the protein content of distinct EV populations is tightly regulated and dependent on the stimulus causing EV release. Apoptosis induction had a significant impact on the protein load of LEVs as well as that of SEVs. However, some proteins (DDB1, EHD3, and GSN) had a high and stable expression within SEVs despite the induction of apoptotic cell death. Thus, we suggest that distinct protein groups might be specific for a particular EV type (e.g., EHD3 and GSN for SEVs). On the other hand, some proteins might be specific for EVs released from T-cells undergoing apoptosis (e.g., proteins of the actin-myosin cytoskeleton, like ACTG1, ACTB, and MYH9). To further substantiate this, we investigated several proteins by western blot analysis. Here, we analyzed membrane associated proteins of the T-cell receptor (TCR) signaling cascade (LAT, LCK, ZAP70, ERK1, and pERK). As shown in Figure 5A, these proteins turned out to be specific for LEVs. Similar results were obtained when we analyzed actin and ezrin as proteins of the actin-myosin cytoskeleton (Figure 5B).

In DIGE analysis, the expression of the proteasome subunits PSMA1 and PSMB4 appeared higher in LEVs when compared to SEVs. Therefore, we investigated subunits of the proteasome also by western blot analysis. As shown in Figure 5C PSMA1 was detected mainly in LEVs and strongly induced after induction of apoptosis (this expression profile matched with the data obtained from DIGE analysis). The proteasome subunit beta-9 (PSMB9) and proteasome subunit beta-10 (PSMB10) were specific for LEVs released from apoptozing T-cells.

A further protein that was detected within apoptotic cell-derived LEVs is the high mobility group box protein B1 (HMGB1). HMGB1 (a nuclear protein involved in structural DNA organization) can be released from necrotic cells or upon cellular activation. After this release into the extracellular space, HMGB1 serves as a mediator of inflammation (49). Interestingly, HMGB1 accumulated nearly exclusively within apoptotic cell-derived LEVs and seemed to be excluded from other EV subpopulations (Figure 5C).

We then analyzed cytosolic/endosomal proteins which have classically been used as exosome markers. These included the proteins TSG101 and HSP70, as well as the analysis of protein ubiquitination. When analyzing SEVs released from viable T-cells, TSG101, and HSP70 as well as ubiquitination of proteins appeared rather specific for isolated SEVs. However, after the induction of apoptosis, TSG101 and HSP70 were found mainly in LEVs, while protein ubiquitination was to be observed in both EV populations (Figure 5D).

Finally, as we had already observed a specific expression of the microtubule-associated proteins GSN and EHD3 in SEVs, released from activated as well as from apoptozing T-cells, we were able to validate these results by western blot analysis (Figure 5E). Thus, these proteins can be used as specific markers for SEVs even after the induction of apoptotic cell death.

### The Proteasome Is Involved in the Regulation of LEV Release

**Figure 6:**
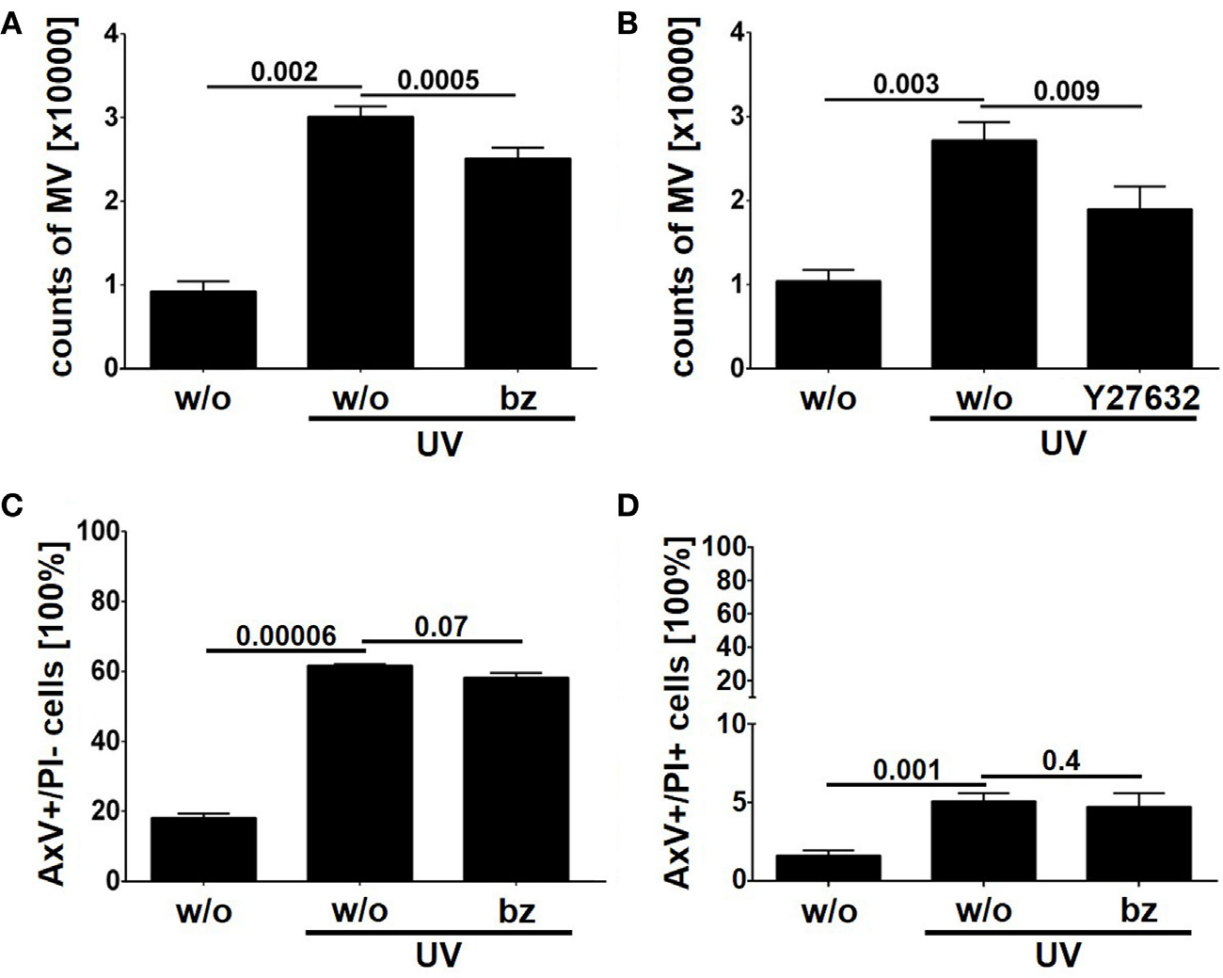
Release of large EVs (LEVs) is impaired by bortezomib. Activated T-lymphocytes were irradiated with UV-B irradiation (90 mJ/cm^2^) to induce apoptosis in presence of 1 nM bortezomib and 10 µM Y27632. After 20 h the amount of released vesicles was analyzed by flow cytometry. **(A)** The amount of released vesicles is shown in the graph. Mean values + SEM are shown. Vesicle release was inhibited by bortezomib. **(B)** The amount of released vesicles is shown (mean values + SEM). Y27632 was used to inhibit LEV release. **(C)** The graph shows the percentage of apoptozing T-cells (analyzed by AxV/PI staining). **(D)** The graph shows the percentage of necrotic T-cells (AxV+/PI+, analyzed by AxV/PI staining). Data were obtained from four independent experiments and statistical significance was calculated employing the Student’s *t*-test.

As described above we detected the proteasome subunits PSMA1, PSMB4 within LEVs. Moreover, we could detect the functional catalytic subunits, PSMB9 and PSMB10 exclusively in LEVs released after apoptosis induction (see Figure 5C). Thus, we questioned whether the proteasome might play a role in the release of LEVs from apoptozing T-cells. To analyze this, T-cells were induced to undergo apoptosis in the presence of the proteasome inhibitor bortezomib. The amount of released LEVs was then quantified by flow cytometry. In parallel, we analyzed the LEV release from apoptozing T-cells treated with Y27632, a rho-kinase inhibitor known to prevent the budding of the plasma membrane and the release of LEVs (23, 50–52). FACS analyses showed a significant increase of LEV release after apoptosis induction, which was significantly reduced in the presence of bortezomib (Figure 6A) as well as in the presence of Y27532 (Figure 6B). Importantly, the total amount of apoptozing T-cells (AxV+/PI−) was not affected by the bortezomib treatment (*p* = 0.07, Figure 6C) and we did not observe any changes in the amount of necrotic T-cells (AxV+/PI+, *p* = 0.4, Figure 6D). Thus, blocking of the proteasome function in fact reduced the release of LEVs from apoptozing T-cells.

## Discussion

The EV research field has been rapidly growing during the past years and various EV subtypes have been described. To date, the heterogeneity of EVs is a well-known fact and it has been shown that SEVs (often called exosomes) as well as LEVs (often referred to as microvesicles or apoptotic bodies) consist of diverse EV subpopulations (5, 17, 37, 39). LEVs are mainly released from the cellular surface and distinct LEV subtypes (such as microvesicles and apopototic bodies) share similar release mechanism (e.g., blebbing of the outer cellular membrane). The discrimination between different LEV populations, however, is very difficult and well-defined discrimination markers are needed.

Distinct EVs subtypes are released in response to a variety of stimuli (5, 38) and it seems likely that a distinct stimulus can trigger the release of a typical EV subtype. As a first of its kind, in this study we systematically analyzed and compared different EV populations released either from activated or from apoptozing human T-lymphocytes.

Isolation of EVs was done by filtration followed by differential ultracentrifugation at 10,000 and 100,000 × *g*. We were able to isolate two EV populations (LEVs and SEVs) from activated or apoptozing T-cells. As expected these EV populations clearly differed in size (Figure 1) and apoptosis induction turned out to be the most potent stimulus for the release of LEVs (Figure 2). Interestingly, the amount of protein per vesicle was diminished after the induction of apoptosis when analyzing LEVs (Figure 2C). This might be explained by diminution of protein synthesis and the degradation of proteins during apoptotic cell death (e.g., caspase-dependent cleavage of proteins).

**Figure 7:**
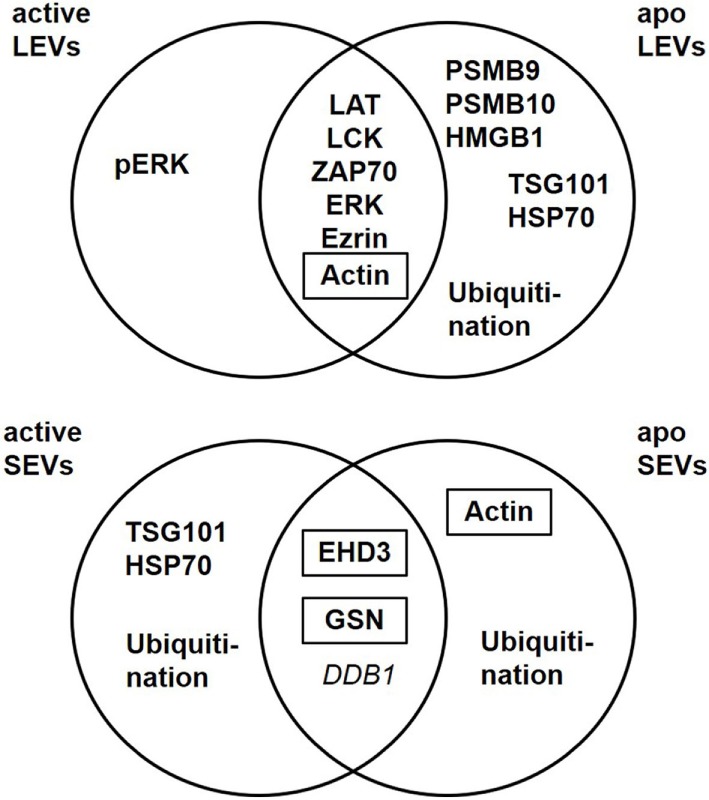
Extracellular vesicles (EV) subtypes are characterized by distinct protein profiles. The upper diagram indicates the distribution of proteins characteristic for large EVs (LEVs), comparing activated and apoptotic LEVs. The diagram below shows the distribution of proteins characteristic for small EVs (SEVs) comparing activated and apoptotic SEVs. Proteins identified by Western blot analysis are shown in bold, proteins identified by western blot as well as mass spectrometry are shown in bold plus frame. Proteins identified by mass spectrometry are shown in italics.

Beside their characteristic differences in size we were also able to demonstrate, that each EV population carries specific proteins, schematically summarized in Figure 7. LEVs released from activated T-lymphocytes carried proteins associated with TCR signaling (like LAT, LCK, ZAP70, ERK1, and pERK) and proteins related to the actin-myosin cytoskeleton (actin and ezrin). SEVs released from activated T-lymphocytes carried the cytosolic and endosomal proteins TSG101 and HSP70, proteins related to the microtubule apparatus (EHD3 and GSN), and the protein DDB1. These observations support the idea, that LEVs are mainly released by budding of the plasma membrane, while SEVs are mainly released from intracellular compartments.

Importantly, the protein content of each vesicle population was dependent on the release stimulus and we observed dramatic changes in the protein profile of LEVs and SEVs after apoptosis induction. While LEVs released after apoptosis induction also carried signaling proteins as well as the cytoskeleton-related proteins, the protein pERK appeared to be rather specific for LEVs released from activated T-cells. We observed an accumulation of the proteasome subunits PSMB9 and PSMB10 exclusively within apoptotic LEVs. Another protein, which was highly specific for apoptotic LEVs, was the molecule HMGB1. This protein is a well-known danger signal, when released into the extracellular space (49). Thus, the packing of HMGB1 into membrane coated vesicles released from apoptotic T-cells might be crucial for the non-inflammatory response to apoptosis within multicellular organisms (53). This was supported by previous observations of our group, having shown, that apoptotic cell-derived membrane vesicles were engulfed by professional phagocytes without the induction of an inflammatory immune response (23, 35, 54). The release of proteasome subunits within *microvesicles* or so-called *apoptotic exosome-like vesicles* has been described previously (55, 56). Nevertheless, we herein demonstrate that apoptosis induction is a specific stimulus for the translocation of the inducible proteasome subunits, PSMB9 and PSMB10, and the danger signal protein HMGB1 into LEVs. For the first time, we present a set of proteins (PSMB9, PSMB10, and HMGB1), which were highly specific for LEVs released after the induction of apoptosis. These proteins can be used to differentiate between apoptotic cell-derived LEVs (or apoptotic bodies) and other LEV subpopulations. Moreover, we have shown that PSMB4, PSMB9 and PSMB10 are specific cargo of LEVs and cannot be found within the SEV population. Based on these findings we were interested, whether the proteasome function is also involved in the regulation of LEV release after apoptosis induction. Using the proteasome inhibitor bortezomib, we were able to demonstrate an involvement of the proteasome in the regulation of LEV release from T-cells induced to undergo apoptosis (Figure 6). Further studies will be needed in order to gain a deeper insight into the proteasomes role as a regulator of apoptotic cell blebbing and the subsequent release of LEVs (or apoptotic bodies).

When analyzing the classically used exosomal marker proteins TSG101 and HSP70 in EVs released from apoptozing T-cells we obtained very interesting results. As described above, TSG101 and HSP70 were specific for SEVs when analyzing EVs released from activated T-cells. In contrast, both proteins accumulated within the LEV fraction after the induction of apoptosis. These results are in line with some other studies, which have shown that TSG101 can be recruited to the plasma membrane and then be released within microvesicles by direct plasma membrane budding (57, 58). Only recently, another study demonstrated the release of HSP70 not only within SEVs, but also within LEVs isolated by low speed centrifugation. In this study, EVs released from human dendritic cells were analyzed (39). We demonstrate here for the first time that apoptosis induction causes the release of TSG101 and HSP70 within LEVs. Moreover, TSG101 and HSP70 are rather excluded from the SEV fraction after the induction of apoptotic cell death.

Ubiquitination has been reported to be crucial for the incorporation of proteins into exosomes (59). Thus, we analyzed the ubiquitination profile of proteins within EVs released from activated or apoptozing T-cells. Analyzing LEVs and SEVs released after cellular activation, ubiquitinated proteins were exclusively present within the SEV fractions. In contrast to this, both, SEVs as well as LEVs contained ubiquitinated proteins when EVs were isolated from apoptozing T-cells.

Beside the changes of the EV protein cargo after apoptosis induction we identified proteins specific for SEVs, regardless of the underlying release stimulus. We detected the proteins EHD3, GSN, and DDB1 in all SEV preparations. Interestingly, EHD3 and GSN have been reported to be regulators of intracellular vesicle trafficking by interacting with the microtubule apparatus (60, 61). Moreover, other members of the EHD family (EHD1 and EHD4) have been identified as SEV-specific proteins in a recent study (39). Based on these findings EHD3 and GSN can be useful markers for the identification of SEVs which are most likely released from intracellular compartments.

Taken together, we identified several proteins which are specific for distinct EV populations released from activated or apoptozing T-cells. LEVs are characterized by proteins which are associated with the actin-myosin cytoskeleton or signaling proteins linked to the cellular membrane. We further demonstrated that proteasome subunits are present in LEV and that proteasomal function participates in the regulation of LEV release from apoptozing T-cells. SEVs, in contrast, carried proteins linked to endosomal and cytosolic compartments and some of these proteins have previously been shown to regulate intracellular vesicle trafficking. We observed substantial change of the EV protein cargo after apoptosis induction. Our findings are very important as apoptotic cell death is present in virtually all tissues as well as in cell culture conditions. Thus, when analyzing EVs, the amount of apoptozing T-cells must always be considered and some of the proteins identified here (Figure 7) may be useful to identify EVs released from apoptozing T-cells. Our data present an important contribution to the EV field as they provide tools for a better characterization of lymphocytic EVs. Moreover, they will substantially help to standardize isolation procedures, improve purity of EV isolates, and enable comparability of various experimental projects dealing with EVs.

## Ethics Statement

This study was carried out in accordance with the recommendations of “ethics guidelines of our institution, ethics committee of the University of Heidelberg” with written informed consent from all subjects. All subjects gave written informed consent in accordance with the Declaration of Helsinki. The protocol was approved by the “ethics committee of the University of Heidelberg.”

## Author Contributions

CT, KB, PS, and MS were participated in concept and research design. CT, KB, PS, LC, and CB conducted all experiments. CT, KB, PS, and MS analyzed data. CT, KB, PS, CB, LC, MS-C, NB, H-ML, and MS drafted and revised the work and critically discussed data for important intellectual content. CT, KB, PS, CB, LC, MS-C, NB, H-ML, and MS wrote or were contributed in writing the manuscript and had a final approve of the version to be published. All authors agreed to be accountable for all aspects of this work.

## Conflict of Interest Statement

The authors declare that the research was conducted in the absence of any commercial or financial relationships that could be construed as a potential conflict of interest.
