# Supplementary material for: Extracellular Vesicle Subtypes Released From Activated or Apoptotic T-Lymphocytes Carry a Specific and Stimulus-Dependent Protein Cargo
Source: Front Immunol. 2018 Mar 15;9:534. doi: 10.3389/fimmu.2018.00534 (PMC5862858; doi:10.3389/fimmu.2018.00534)
Supplement: Supplementary file 1 [file presentation_1.pptx]

## Slide 1
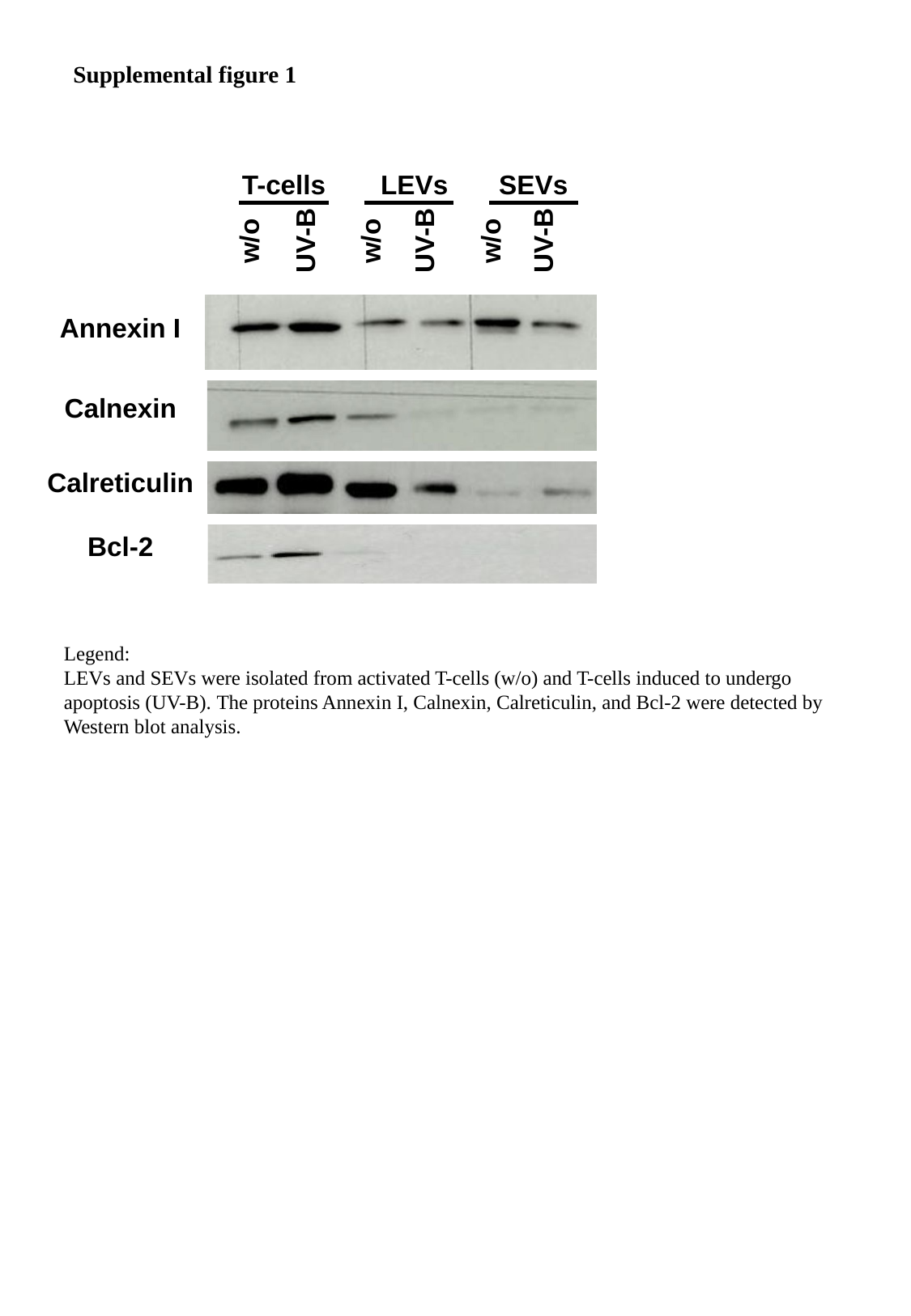

Supplemental figure 1
T-cells
LEVs
SEVs
UV-B
UV-B
UV-B
w/o
w/o
w/o
Annexin I
Calnexin
Calreticulin
Bcl-2
Legend:
LEVs and SEVs were isolated from activated T-cells (w/o) and T-cells induced to undergo apoptosis (UV-B). The proteins Annexin I, Calnexin, Calreticulin, and Bcl-2 were detected by Western blot analysis.
